# Effectiveness of structured education and training in perineal wound assessment and repair for midwives and midwifery students: A review of the literature

**DOI:** 10.18332/ejm/134511

**Published:** 2021-05-20

**Authors:** Monica P. Diaz, Naomi Simpson, Angela Brown, Faith C. Diorgu, Mary Steen

**Affiliations:** 1 UniSA Clinical and Health Sciences, University of South Australia, Adelaide, Australia; 2 Department of Nursing Science, Faculty of Clinical Sciences, University of Port Harcourt, Port Harcourt, Nigeria

**Keywords:** perineal trauma, childbirth, midwives, education, confidence and skills

## Abstract

**INTRODUCTION:**

Perineal trauma is a commonly observed complication of childbirth, affecting more than 75% of women who have a vaginal birth. Perineal trauma is associated with significant short- and long-term comorbidities that negatively impact women’s quality of life. Severe perineal trauma (SPT) rates in Australia have almost doubled in the last decade. Reasons for increased rates are not completely understood; some researchers suggest improvements in diagnosis and reporting, while others have a view that it may be due to a lack of structured and standardized education in perineal wound assessment and repair for clinicians.

**METHODS:**

The Joanna Briggs Institute (JBI) scoping review methodology was adopted as a systemic process to identify studies that have investigated the effectiveness of perineal wound assessment and repair education and training for midwives and midwifery students.

**RESULTS:**

Five studies met the inclusion criteria for this review, to have evaluated a type of education or training, on childbirth-related perineal wound assessment and repair that included midwives and midwifery students. A total of 1279 midwives and midwifery students volunteered to participate in all five studies. The length of the education or training implemented varied between each study from a 1-day workshop to 100 hours of education. All five studies measured the effectiveness of each program through changes in participants’ confidence, knowledge and skills in perineal assessment and repair before and after an intervention using various self-assessment questionnaires.

**CONCLUSIONS:**

The implementation of a structured educational workshop on perineal wound assessment and repair improves the confidence, skills and knowledge of midwives and students.

## INTRODUCTION

Perineal trauma is defined as any injury to the genitalia sustained during a vaginal birth, which can occur spontaneously or deliberately, as a result of an episiotomy^1^. Perineal tears are classified from first to fourth degree, depending on the anatomical structures involved in the damage^2^. Severe perineal trauma (SPT) refers to 3rd and 4th degree tears where the injury extends to the external and internal anal sphincter, respectively^3^. These injuries, if misdiagnosed and inadequately repaired, can have significant short- and long-term negative consequences for women including: faecal and urinary incontinence (in approximately 60–80% of cases), perineal pain, dyspareunia and depression^4-6^. Furthermore, perineal trauma can also affect maternal attachment with their newborns and relationships with their partners/family^1^. The consequences of perineal trauma cause a financial burden on health systems^7^, which may be minimized through ongoing education around the risks, recognition and management of perineal trauma^1^.

A systematic review by Morris et al.^8^ found that there are significant gaps among midwives and obstetricians’ perceived knowledge of perineal trauma assessment and classification. This has considerable implications in practice, as a poor understanding of perineal anatomy, assessment and repair, leads to the misclassification and the inadequate repair of perineal trauma^8^. A lack of anatomical knowledge of perineal structures, as well as poor systematic assessment of perineal tears, results in approximately 33% of third- and fourth-degree tears incorrectly classified or completely missed at the time of repair^9-11^. Midwives and obstetricians attribute this knowledge deficit to a lack of structured educational training in undergraduate programs and clinical practice^12-14^. Midwives are at the frontline when it comes to preventing, diagnosing and repairing childbirth related perineal trauma. Yet, many do not have the training or confidence to assess and repair childbirth related perineal trauma despite it being within their scope of practice^12,13^. The aim of this literature review was to identify studies that have investigated and explored perineal wound assessment and repair educational programs for midwives and midwifery students.

### Review questions

- What is the effectiveness of perineal wound assessment and repair education for midwives and midwifery students?
- How is the effectiveness of perineal wound assessment and repair education for midwives and midwifery students measured?

### Inclusion criteria

A population, concept, context (PCC) framework was used to identify the main subjects and inform the inclusion criteria.

#### Population

This review has included all studies that investigated the effectiveness of perineal wound education/training for midwives and midwifery student populations.

#### Concept

This review considered all studies that investigated the effectiveness of perineal wound assessment and repair education/training. The studies had to include measurements of participants cognitive, or affective, and/or psychomotor skills such as knowledge, confidence and skills.

#### Context

The studies included in this review evaluated a type of education, or course and/or training on childbirth related perineal wound assessment and repair.

### Exclusion criteria

Studies that had specific interventions (such as repair of third- and fourth-degree tears or episiotomies only) or did not provide education in perineal wound assessment and repair; and did not measure the effectiveness of the training for midwives or midwifery students were excluded. The rationale for excluding studies with specific intervention was related to the manner in which repairs of third- and fourth- degree tears are undertaken in Australia. These tears are predominantly managed in an operating theatre by obstetricians or medical officers and midwives are not usually involved in these repairs^15-18^.

### Types of sources

This review considered all experimental and quasiexperimental studies, analytical observational studies and descriptive observational studies. Systematic reviews that met the inclusion criteria were also considered. Theses and dissertations were excluded due to time constraints. Only English language articles were included in this review because of a lack of financial resources to translate articles.

## METHODS

**Figure 1:**
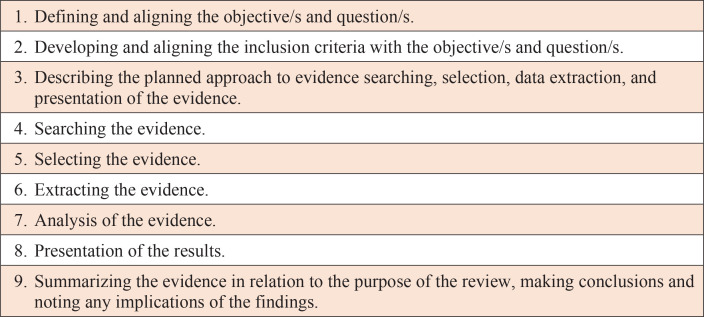
JBI Scoping review framework 2020 enhancements*

The Joanna Briggs Institute (JBI) 2020 scoping review framework was adopted for this critical literature review to ensure a structured search and in-depth review of the literature, that investigates the effectiveness of perineal wound assessment and repair education and training for midwives and midwifery students^19^ (Figure 1).

### Search strategy

The JBI three-step search strategy^19^ was used to search Ovid Emcare and Embase databases initially to identify keywords and index terms from titles and abstracts. A librarian was consulted during this process to ensure that a comprehensive list was obtained. The search terms list was then used across all included databases.

### Information sources

A literature search was performed across six primary and secondary electronic databases including Medline, Ovid Emcare, Embase, Joanna Briggs Institute of Evidence Based Practice, Wiley Online Library, and The Cochrane library. A search of Google scholar, and ‘hand-searching’ the reference lists of journal articles was also conducted and used to identify appropriate research studies.

**Figure 2:**
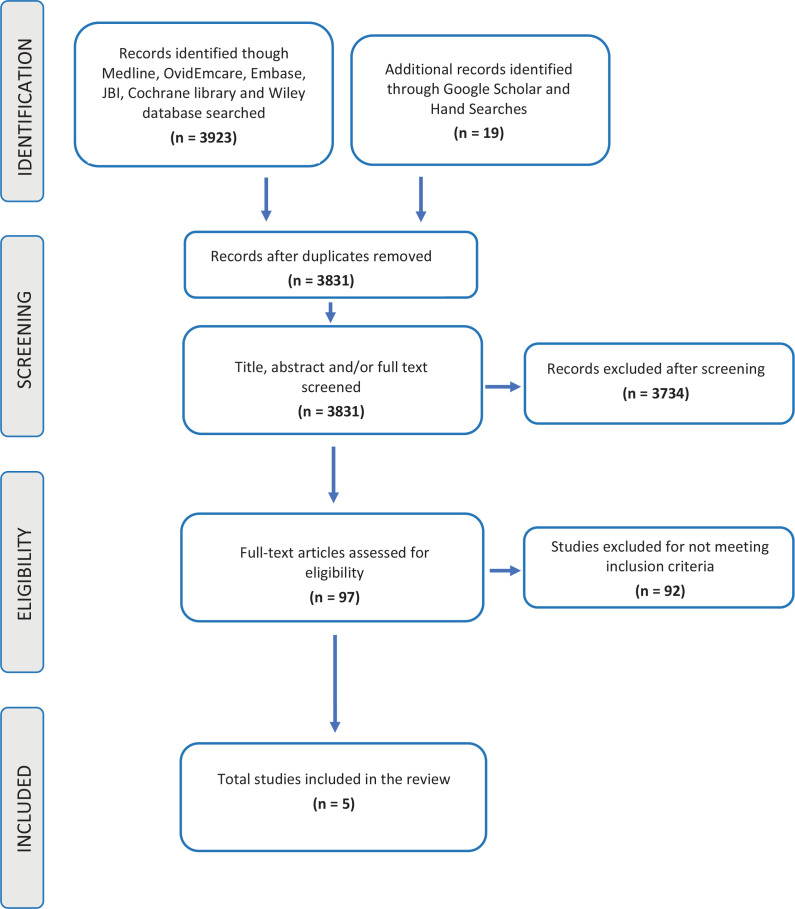
PRISMA flowchart summary of search strategy and study selection

Using the key search terms and relevant synonyms, the most productive search string was: [Perineum (OR perineum* OR perineal) AND Education (Educat* OR learn* OR teach* OR activit* OR program* OR train* OR workshop* OR seminar* OR simulation*) AND {Midwifery OR Midwives (midwi* OR nurse* OR birth* OR attendant*)}]. The publication date was open and included journal articles, textbooks, websites, primary research studies, systematic reviews, letters, guidelines, and meta-analysis^19^. The search of the six bibliographical databases was performed during July and August 2020. The final search strategies and results are provided in Figure 2.

### Study selection

Following the final search, all identified citations were collated and uploaded to bibliographic manager EndNote where 111 duplicates were removed. A two-person screening approach of titles and abstracts of the final 3942 search result articles against the inclusion criteria for this review was applied to reduce bias, with a third reviewer consulted to reach consensus. First screening excluded 3734, due to incorrect context and demographic criteria. Full-text screening of 97 articles was attempted by two of the reviewers. A total of 94 full-text articles were retrieved and three full-text articles were not available. No further attempts were made to access the full texts of the three articles not available as the description in the abstracts did not appear to meet the inclusion criteria. Following full-text screening, 5 articles met the inclusion criteria.

The resulting 5 articles were individually analyzed using the CASP cohort studies checklist, to determine the studies credibility, dependability, confirmability, transferability and authenticity using a rigorous approach (Table 1)^20,21^.

**Table 1:** CASP cohort study checklist summary of screened articles

	*Andrews et al.^23^ 2005*	*Selo-Ojeme et al.^24^ 2009*	*Wilson^22^ 2012*	*Zimmo et al.^14^ 2017*	*Diaz et al.^25^ 2020*
1. Did the study address a clearly focused issue?	Yes	Yes	Yes	Yes	Yes
2. Was the cohort recruited in an acceptable way?	Can’t tell	Yes	Yes	Yes	Yes
3. Was the exposure accurately measured to minimize bias?	Can’t tell	Yes	Yes	No	Yes
4. Was the outcome accurately measured to minimize bias?	Yes	Yes	Yes	No	Yes
5a. Have the authors identified all important confounding factors?	Yes	Yes	Yes	No	Yes
5b. Have they taken account of the confounding factors in the design and/or analysis?	Yes	Yes	Yes	No	Yes
6a. Was the follow-up of the subjects complete enough?	No	No	Yes	No	Yes
6b. Was the follow-up of the subjects long enough?	No	No	Can’t tell	No	No
7. What are the results of this study?	Following the handson workshop there was an increase in the participant’s ability to classify obstetric anal sphincter injuries accurately and a change in practice relating to their ability to perform a rectal examination before and after perineal repair.	The authors found there was a significant increase in the use of the recommended evidence-based technique for perineal repair 4 months after training (28% vs 100%; p<0.001), and in the mean scores for knowledge and skills in all the domains (p<0.001). Additionally, the participants felt the women were happy with the new technique.	The authors found that midwives’ competency improved following an educational intervention in five intervention Trusts in perineal repair. The comparison Trust demonstrated a non-significant difference. They also found that the education program increased the levels of confidence and competency when assessing and managing perineal repair.	The authors found improvements in knowledge and diagnosis of perineal tears for doctors and midwives after attending a 2-day training workshop.	The inclusion of a perineal wound assessment and repair workshop for undergraduate Midwifery students improved the students’ knowledge and confidence in the management of childbirth related perineal trauma.
8. How precise are the results?	All results except one showed a p<0.05	p<0.001	p<0.05	All results except one showed p<0.05	p<0.05
9. Do you believe the results?	Yes	Yes	Yes	Yes	Yes
10. Can the results be applied to the local population?	No	No	No	No	No
11. Do the results of this study fit with other available evidence?	Yes	Yes	Yes	Yes	Yes
12. What are the implications of this study for practice?	Knowledge of doctors and midwives attending the workshop in perineal anatomy and repair is suboptimal and that hands-on workshops can significantly improve practice. Hands-on education in technical skills on perineal repair should be included in the training programs of doctors and midwives	Hands-on training is an effective way of improving the skills of midwives performing perineal repair and leads to modification of clinical practice.	Perineal repair training can improve midwives’ confidence and competency in perineal repair.	Perineal repair training can improve the knowledge for doctors and midwives in clinical practice.	The inclusion of a perineal wound assessment and repair workshop for undergraduate Midwifery students improved the students’ knowledge and confidence in the management of childbirth related perineal trauma and would be of benefit for undergraduate midwifery programs in Australia.

## RESULTS

A summary of the characteristics of the five studies are presented in Table 2. The characteristic words used to summarize each study were: author, year, country, title of study, aim, sample size, method/tools, findings, and conclusions.

**Table 2:** Characteristics of included studies

*Author Year Country*	*Title*	*Aim*	*Sample size*	*Method/tools*	*Findings and conclusions*
**Diaz et al.^25^ 2020 Australia**	Perineal wound assessment and repair education for midwifery students: a multi-methods study.	To evaluate the effectiveness of perineal wound assessment and repair training for midwifery students in the Bachelor of Midwifery program at a South Australia tertiary institution.	65 midwifery students	Multi-method design with a quantitative pre-post-test and qualitative component. Knowledge and self-confidence were measured using: A multiple-choice questionnaire (10 questions) based on the content of the workshop. A 5-point Likert scale questionnaire and a Student Satisfaction and Self-confidence in Learning (SSSL) questionnaire evaluated students perceived self-confidence with perineal assessment and repair. A qualitative self-reflective journal was used to collect information on the participants’ experiences with perineal trauma during their clinical placements.	45 midwifery students completed the pre-post-test questionnaires and 21 completed the self-reflective journals. Changes in midwifery students’ postworkshop mean test scores for knowledge were statistically significant (p<0.001) demonstrated an overall improvement. Midwifery students’ self-confidence with perineal wound assessment and repair also improved after the educational workshop (p<0.001). Five themes and 12 subthemes were identified from the students’ self-reflective journals. These were: communication is important; confidence growth; perineal pain; perineal outcomes are associated to practitioner’s skills and experience; reflecting on the experience.
**Zimmo et al.^14^ 2017 Palestine**	Diagnosis and repair of perineal injuries: knowledge before and after expert training—a multicenter observational study among Palestinian physicians and midwives.	To assess whether a 2-day training with experts teaching on diagnosis and repair of perineal injuries among Palestinian midwives and physicians could change their level of knowledge towards the correct diagnosis and treatment.	150 participants (64 physicians and 86 midwives)	Prospective multicenter observational study across 6 Palestinian hospitals. A questionnaire with 14 questions on diagnosis and repair of perineal tears was given to all participants before, and 3 months after, a 2-day training program on perineal assessment and repair.	124 (80%) of participants completed the 3 months follow-up questionnaire, 71 of those were midwives. Midwives had the greatest improvement in knowledge regarding perineal anatomy (43.3% improvement), episiotomies (32.7% improvement), assessment and classification of perineal trauma after the training (18%). A lack of systemic postgraduate education may explain variations in perineal tears knowledge prior to the attendance to the workshop.
**Wilson^22^ 2012 UK**	Effectiveness of an educational programme in perineal repair for midwives.	To evaluate the effectiveness of a work-based module and inservice educational programme in perineal repair for midwives on their perceived level of competency undertaking this skill in clinical practice.	208 midwives	A quasi-experimental pre-post intervention case study, combining a non-equivalent comparison group and evaluation action research. Participants from 5 NHS trusts chose between: attending 46 × 2 hour in-service workshops (92 hours) or a work-based module through the Midwifery practice program (100 hours). Comparison group (1 NHS trust) originally had no intervention. Midwives were asked to rate their perceived level of competency (levels 0-6) using the ‘Developing a curriculum’ (DACUM) model in the pre- and post- intervention questionnaires. Independently measured variables consisted of: knowledge, skills and views that represented midwives’ attitudes and values.	Only 145 responded to questionnaires post training. There were no significant differences in midwives’ demographics across the six NHS Trusts. No significant differences in knowledge were found between pre-and post-intervention groups. However, a strong positive correlation of application of new knowledge to practice was noted (p<0.0001). Prior to the intervention 40.3% of midwives felt that they did not need supervision of perineal repair, this increased to 86.2% after the intervention.
**Selo-Ojeme et al.^24^ 2009 UK**	Impact of a structured, hands-on, surgical skills training program for midwives performing perineal repair.	To evaluate the effect of structured hands-on training for midwives performing perineal repair.	719 midwives	Cohort of midwives attended a 1-day workshop on perineal surgical skills. Participants completed an anonymous questionnaire prior to and immediately post the workshop and again at 4 months after the workshop. The questionnaires included a scoring system on a visual analogue scale (VAS) for knowledge and skills in three domains: instrument handling, tying of surgical knots, and subcuticular perineal repair.	251 midwives had performed perineal repair in the past and over 70% of participants used interrupted technique for suturing prior the training. 151 midwives (50.3%) completed the follow-up questionnaire at 4 months. Four months after training none of the participants used interrupted technique. Mean scores for knowledge and skills in the three domains significantly improved immediately after the workshop and were maintained after 4 months. Midwives who worked in labor ward or birth centers were more likely to perform repairs than those working in other areas and had higher mean score for knowledge (p=0.007), skills of knot tying (p=0.01) and continuous subcuticular repair (p=0.003).
**Andrews et al.^23^ 2005 UK**	Can hands-on perineal repair courses affect clinical practice?	To determine whether attending a hands-on training workshop in repair of episiotomy and second-degree tears changed clinical practice by increasing participants’ ability to accurately classify obstetric anal sphincter injuries (OASIS) and manage perineal trauma according to best practice.	208 participants (3 midwifery students, 198 midwives, 7 junior doctors)	Pre-test, post-test study. Participants were asked to complete a questionnaire comprising 14 questions on perineal injury assessment and repair prior to a 1-day hands-on workshop. The same questionnaire was sent to all participants to complete, 8 weeks after the workshop.	Only 147 participants completed the follow-up questionnaire at 8 weeks after the workshop. There were significant improvements in the knowledge of midwives and doctors regarding the classification of OASIS after the workshop. Performing a rectal examination prior to repair improved by 61% after the workshop. 133 participants worked in units with protocols for management of 2nd degree tears. 24% of participants felt their training was poor at the time of their first unsupervised repair and 40% felt that their training could be improved. Changes to techniques adopted for performing perineal repair also improved significantly (p<0.001).

### Included studies

Three of the studies were conducted in the United Kingdom^22-24^, one in Palestine^14^, and one in Australia^25^. The targeted population groups in two of the studies were doctors and midwives^14,23^, whilst two studies only included midwives^22,24^ and one included midwifery students only^25^. A total of 1279 midwives and midwifery students volunteered to participate across all five studies.

All included studies used either a purposive and/or convenience sample of participants^14,22-25^. All five studies lost a number of participants due to attrition. One of the studies target population included doctors and midwives^14^, two studies included midwives only^22,24^, one study included doctors, midwives and midwifery students^23^, and one study included midwifery students only^25^. Participants were reported to have varying perineal assessment and repair experience^14,22-25^.

All five studies employed a similar research methodology, i.e. the administration of a pre-test and post-test questionnaire or assessment, before and after the attendance at an educational workshop on perineal wound assessment and repair.

The length of the intervention in each of the studies varied from a one- to two-day workshop^14,23-25^ to 100 hours of education^22^. The programs all included lectures, videos and hands-on skills training on high fidelity models. Two studies mentioned that the information included in the lectures adhered to current clinical guidelines^22,24^. Diaz et al.^25^ was the only study that provided an overview of the content of the education which was designed around ‘risk, recognition, repair and relief’ of perineal wound management as identified by Steen^26^. The authors of this included article^25^ also stated that the content included ‘perineal anatomy and current evidence for perineal care and healing’, however, there are no references to indicate what sources were used to support current evidence.

### Effectiveness of perineal wound assessment and repair education for midwives

Each of the five studies measured the effectiveness of education through one domain or a combination of three domains – confidence, knowledge and skills – which have been identified in the literature as essential to the success of perineal repair^12,13^. The strengths and limitations of each study are presented in Table 3.

**Table 3:** Strength and limitations of included studies

*Study*	*Strengths*	*Limitations*
**Diaz et al.^25^ 2020**	Multiple data collection tools. Description of study design provided. A multi methods design and validated assessment tools were used. A pilot study was completed. Ethics was discussed. Addition of the journal entries from students was a strength.	Low number of participants completed the follow-up multiple choice questionnaire at 4 months (5%). Information bias may be an issue as questionnaires were self-reported. Longer term effects (e.g. 12 months) of the training were not reported. Cost of the intervention not-- discussed.
**Zimmo et al.^14^ 2017**	Description of study design given. Ethical approval discussed. Silicon models of human sphincters and animal sphincters were used in the training to give students a more realistic environment to practice their skills. 83% response rate in follow-up questionnaire.	The practical component of the training was not evaluated. Information bias may be an issue as questionnaires were self-reported. The questionnaire was not given to participants immediately after training therefore, any loss of knowledge could not be determined at the 3 months follow-up questionnaire. The areas midwives worked in was not reported (e.g. antenatal, intrapartum or postnatal). This could have some bearing on the results collected 3 months post training as a midwife working in an intrapartum setting is more likely to continue practicing the skills attained during training than a midwife working in an antenatal setting. Longer term effects (e.g. 12 months) of the training were not reported. No discussion provided on the cost of the intervention.
**Wilson^22^ 2012**	Description of the study provided. Many educational opportunities were provided to participants. Participation of six midwifery/consultant led NHS trust hospitals in South East England. Ethics discussed. Results summarized well. Discussion relevant to the aims of the research.	Difficult paper to read. The information could have been divided into two articles. Lots of interventions implemented. It would be difficult to replicate as a lot of time would need to be invested by participants. Differences between the 6 NHS trust hospitals not discussed. No indication of anonymity of questionnaires. Midwives’ perceived knowledge could not be attributed to one intervention as there were a number of factors (external study days, private study and peer group learning) that may have affected the outcomes. Comparison group lost during study. Poor response rates in follow-up questionnaire. No discussion on the cost of this intervention.
**Selo-Ojeme et al.^24^ 2009**	The one-day workshop included lectures, audio-visual demonstrations and hands-on training. Skin pads and oxtongues were used in the skills training giving participants a more realistic feel for the repair.	No mention of recruitment process or where cohort of midwives came from. Self-reported questionnaire could be subject to response bias. The anonymity of the questionnaire made it impossible to follow up any individual changes. Low number of participants completed the follow-up questionnaire at 4 months. No ethics discussed. No cost provided on the intervention.
**Andrews et al.^23^ 2005**	The 1-day workshop comprised lectures, videos and hands-on training on foam pads and latex perineal models. Good arguments in the discussion. Aim of the study highlighted. The questionnaire used in the study is presented in the article.	All data were analyzed together so differences between midwifery students, midwives and junior doctors could not be determined. It was not reported whether the questionnaires were anonymous or not. Skills were not tested. Individual changes in knowledge and skill were not analyzed. Information bias may be an issue as questionnaires were self-reported. The questionnaire was not completed immediately after the workshop, but 8 months later so immediate effectiveness of the training was not measured. Cost of the intervention not discussed.

#### Confidence

Improvements in participants’ self-confidence on knowledge and skills in perineal repair after the implementation of an educational workshop were reported by Wilson^22^ and Diaz et al.^25^.

Midwives in the study of Wilson^25^ reported to feel more confident with the knowledge on perineal assessment and repair after receiving education than they did prior. The midwives also reported to feel more confident with their skills in undertaking perineal repair when supported by an experienced perineal repair supervisor. This was most noted among midwives who worked in birthing suites as they had more opportunities to practice than those in other areas. Diaz et al.^25^ found that median post-workshop scores of midwifery students’ self-reported confidence in anatomy and physiology of the perineum, assessment and recognition of perineal trauma, and repair, were statistically higher (p<0.0001) than those reported pre-workshop, further supporting the findings of Wilson^22^.

#### Knowledge and skills

All five studies found statistically significant improvements in midwives’ and midwifery students’ knowledge of perineal anatomy, assessment and repair after attending an education workshop^14,22-25^. Andrews et al.^23^ found that participants’ knowledge on the classification of anal sphincter trauma improved significantly after a one-day ‘hands-on’ workshop. This was also true for conducting a rectal examination before beginning perineal repair (28% pre-workshop vs 89% post-workshop, p<0.001), and best practice for suturing perineal muscles (i.e. continuous suture) (32% pre vs 84% post, p<0.001) and skin layer (i.e. subcuticular suture) (39% pre vs 81% post). Zimmo et al.^14^ used a similar questionnaire implemented in the Andrew et al.^23^ study and found comparable results: knowledge of anal sphincter trauma classification (9.8% pre vs 54.2% post, p<0.001); rectal examination prior to suturing (0% pre vs 18% post, p<0.001); and using subcuticular suture for skin repair (26.1% pre vs 50.7% post, p<0.001), after a two-day workshop.

Diaz et al.^25^ found that midwifery students’ mean test scores post-workshop (mean=8.8, SD=1.1) on knowledge of perineal anatomy, classification of perineal trauma, perineal trauma risk assessment, episiotomy and perineal repair were statistically significantly higher than pre-workshop mean test scores (mean=6.9, SD=1.6). Selo-Ojeme et al.^24^ found that participants’ self-rating mean test scores on their knowledge and skills in instrument handling, knot tying, and recommended technique to employ for repair, were also statistically significant immediately and four months after the workshop. Wilson^22^, on the other hand, did not find a significant difference in the perceived knowledge of midwives’ management of perineal trauma, but did find a positive correlation (r=0.647; 102; p<0.000) in the transfer of new knowledge to clinical practice. These findings are supported by Diaz et al.^25^ who found that midwifery students were able to use the information learnt during their educational workshop to reflect on clinical practice of perineal assessment and repair, as reported in their personal reflective journals.

## DISCUSSION

The results reported in each included study demonstrate that the introduction of an educational program in perineal assessment and repair is effective in improving knowledge, confidence and skills of participants^14,22-25^.

The inclusion of various learning modalities (e.g. text, audio-visual and hands-on) in all the training programs is a strength for all the studies, as it demonstrates the embracement of multimodal (visual, auditory, read and write and kinaesthetic) types of learning preferences of individuals^27^. These findings are consistent with research in simulation and skills training as successful methods of teaching clinical skills to midwives^28-31^. Wilson et al.^22^ used a number of interventions to educate midwives on perineal assessment and repair. A choice was given to participants of attending 46 two-hour in-service education sessions (92 contact hours) or enrolling in a work-based module through a Midwifery practice program (100 hours) for the duration of the study (2004–2005), however, the results between the two groups were not compared and no reason for this was provided. This type of intervention would have meant a significant commitment was required from the participants and their employers (the in-services took place in the hospital setting). This study could be costly and difficult to replicate in practice, as a 1-day or 2-day workshop, like those provided in the other three studies^14,23,24^, required less time from the participants and achieved the same outcomes as those noted by the Wilson et al.^22^ study. However, this is only an assumption, as the cost of the interventions were not discussed by any of the authors making it difficult to know whether any would be cost-effective in clinical practice.

The length of time that lapsed between the intervention and follow-up questionnaires varied significantly between each study (8–16 weeks), as such, it is difficult to know whether the intervention was the reason for the results or this was the effect of other types of learning that had occurred outside the intervention. Selo-Ojeme et al.^24^ and Wilson^22^ identified that confidence in perineal repair correlates to the number of opportunities available to practice the skills. In other words, midwives who worked in labor wards were reported to have higher levels of knowledge, confidence and skills in perineal repair than those that worked in other areas (such as antenatal and postnatal wards), as they had more opportunities to practice their skills. These findings are supported by research in other areas, which have found that the long-term maintenance of skills is dependent on the ability to practice those skills^32-35^. In the context of translating these findings to practice, opportunities to practice assessment and perineal repair skills can be created either through rotation in labor wards or regular opportunities to attend clinical updates in practice^22,24^.

The response level in follow-up questionnaires varied significantly across the studies, from 50.3%^24^ to 80%^14^ demonstrating a transfer bias. Diaz et al.^25^ reported to have lost 95% of the participants in the follow-up test at four months, the most in any of the studies. The authors attributed this loss to the follow-up questionnaire taking place right at the end of midwifery students’ final year of study, therefore, most would have been pre-occupied completing their last assessments. However, this did not impact the overall findings as the researchers also collected qualitative data through a self-reflective journal from the participants to reflect on the translation of theory to practice during their clinical placements eight weeks after the intervention^25^. Diaz et al.^25^ also found that although midwifery students were not able to perform perineal repair, they were able to conduct assessments of perineal trauma under supervision. Students’ self-reflective journals revealed that the workshop provided them with the knowledge needed to conduct perineal assessments and recognize the severity of perineal injuries. The study by Zimmo et al.^14^ had the highest response rate which could be attributed to the distribution by hand of the questionnaire to each participant at three months after the study. Although time consuming, this was shown to be more effective than the methods implemented by any of the other studies (e.g. mailed questionnaires).

Zimmo et al.^14^, Selo-Ojeme et al.^24^ and Andrews et al.^23^ all identified that a flaw in their design was the use of self-reported questionnaires; Diaz et al.^25^ and Wilson^22^ did not. It is often argued that self-reported questionnaires could be unreliable and compromised by self-reporting bias^36^. Bias in research is defined as ‘any systematic error in the design, conduct or analysis of the study’^37^. It can arise for a number of reasons, in the case of these studies social desirability and recall bias could have played a part in the results^38^. Social desirability is a form of internal bias that arises from the desire of social acceptance and approval^37^. With the exception of the study by Andrews et al.^23^, all recruited participant groups were either midwives, student midwives, or a mixture of midwives and doctors, working in the same institutions. It could be argued that participants knew each other or knew the researchers and, therefore, could have altered their answers so their responses could appear more ‘desirable’. However, given that these studies were looking at the effectiveness of an educational workshop, it could also be argued that a self-reported questionnaire was the best option for measuring the domains tested (e.g. knowledge and confidence)^39^. One way of reducing this form of bias would be to use an anonymous questionnaire such as the one used by Selo-Ojeme et al.^24^, however, the limitation of this is that it is difficult to follow up individual changes.

Andrews et al.^23^, Zimmo et al.^14^, and Diaz et al.^25^, discussed the lack of validity and reliability of their questionnaire tool as a limitation in their research. This is a limitation of the research design process as testing the validity and reliability of the data collection instruments ensures that they measure what they are supposed to and produce the same results under the same conditions at different points in time, thus reducing random error and bias of the results^40^. A lack of validity and reliability of data collection instruments can also render a study ‘weak’, as it is difficult to know whether the results are an accurate measure of the intervention or a product of the instrument used to collect the data^41-44^.

Wilson^22^ and Diaz et al.^25^, designed and piloted the tools for their studies, however, no examples were provided about the kind of questions that were included in the semi-structured questionnaires, therefore, it is not possible to comment on the construct validity of these instruments. On the other hand, Selo-Ojeme et al.^24^ summarized their questionnaire in tables that included the results for each question, making it easier to understand what was being measured. Selo-Ojeme et al.^24^ also used a visual analogue scale (VAS) in their questionnaire where participants self-scored their knowledge and skills on three domains: instrument handling, tying surgical knots, and subcuticular perineal repair^24^.

We summarize that:

- The implementation of educational workshops in perineal wound assessment and repair improves the confidence, knowledge and skills of midwives and student midwives;
- Structured hands-on skills training that includes multimodal learning is effective for teaching perineal repair skills;
- More opportunities to practice perineal assessment and repair skills leads to increased levels of confidence in midwives;
- One-day workshop is as effective as a two-day workshop to teach perineal wound assessment and repair skills; and
- Knowledge and skills are retained from this type of education up to six months post the intervention.

## CONCLUSIONS

This literature review has highlighted that a structured hands-on workshop, for assessment and repair of perineal wounds that incorporates multimodal learning, appears to improve midwives’ and midwifery students’ knowledge, confidence and skills. In the context of analyzing each included study individually, all had a number of strengths and limitations, as well as methodological flaws. However, when examined collectively, the flaws and limitations are outweighed by the strengths and findings that demonstrate the benefits of educational programs in perineal wound assessment and repair for midwives. Future research on perineal trauma assessment and repair education for midwives needs to address what evidence-based content is most essential to include in this type of education that aligns with clinical policies and that is cost-effective, in order to reduce perineal trauma rates globally and the associated morbidity.
